# Effect of high-dose intravenous vitamin C therapy on the prognosis in patients with moderately severe and severe acute pancreatitis: protocol of a prospective, randomized, double-blinded, placebo-controlled study

**DOI:** 10.3389/fmed.2023.1278167

**Published:** 2023-11-09

**Authors:** Wenwu Sun, Bing Zhao, Jiaoyan Li, Yihui Wang, Xing Qi, Ning Ning, Silei Sun, Mengjiao Li, Yi Yao, Tongtian Ni, Li Ma, Juan He, Jun Huang, Zhitao Yang, Ying Chen, Huiqiu Sheng, Enqiang Mao

**Affiliations:** ^1^Department of Emergency, Ruijin Hospital, Shanghai Jiao Tong University School of Medicine, Shanghai, China; ^2^Department of Pharmacy, Ruijin Hospital, Shanghai Jiao Tong University School of Medicine, Shanghai, China; ^3^Department of Cardiovascular Medicine, State Key Laboratory of Medical Genomics, Shanghai Key Laboratory of Hypertension, Shanghai Institute of Hypertension, Ruijin Hospital, Shanghai Jiao Tong University School of Medicine, Shanghai, China

**Keywords:** vitamin C, acute pancreatitis, protocol, randomized controlled trials, intravenous infusion

## Abstract

**Introduction:**

Acute pancreatitis is a common gastrointestinal disease. The mortality of patients affected by severe acute pancreatitis (SAP) remains high. It is unclear whether high-dose intravenous vitamin C (HDIVC) therapy could improve the prognosis of these patients. The current prospective, randomized, double-blinded, placebo-controlled study will explore the effect of high-dose intravenous vitamin C therapy on the prognosis in patients with moderately severe and severe acute pancreatitis.

**Methods and design:**

A total of 418 participants with moderately severe and severe acute pancreatitis who meet the eligible criteria will be randomly assigned in a 1:1 ratio to receive treatment with HDIVC (200 mg/kg/24 h) or placebo (saline) for a period of 7 days. The primary outcome is 28-day mortality in these patients. The secondary outcomes include organ functions and interventions, laboratory tests, healthcare, and 90-day mortality.

**Ethics and dissemination:**

This protocol was approved by the institutional ethics board of the Ruijin Hospital, Shanghai Jiao Tong University School of Medicine, Shanghai, China (Registration Number: 2019-90). The report of the study will be published in peer-reviewed journals and presented at conferences, both nationally and internationally.

**Clinical trial registration:**

Chinese Clinical Trial Registry (ChiCTR1900022022). Version 1.5.

## Introduction

Acute pancreatitis is a common gastrointestinal disease, with an annual incidence of 34 per 100,000 person-years (1, 2). Most patients present with a mild clinical course, but ~20% of patients develop moderately severe or severe acute pancreatitis (SAP). Although the therapy improved significantly in the past decades, the overall mortality rate of SAP remains as high as 20–40% globally (3, 4). After the onset of pancreatitis at the early stage, uncontrolled oxidative stress and large amounts of inflammatory cytokine release induce systemic inflammatory response syndrome (SIRS) that contributes to progressive organ dysfunction. Multiple organ dysfunction syndrome (MODS) is the main cause of death in these patients. Compared to the infection-induced MODS in the later stage, uncontrolled oxidative stress-induced MODS is the main cause of death in the early stage (5). Therefore, timely scavenging of excessive oxidative-free radicals is regarded as a crucial action for better prognosis. In human trials, data about the effect of antioxidants on acute pancreatitis remain sparse. Most of the trials are small-scale and the conclusions are elusive (6).

Vitamin C (VC) is an important antioxidant and performs many physiological functions in the human body. Under normal physiological conditions, plasma VC concentration is ~80 μM. VC is a fast-acting antioxidant in the plasma and could effectively detoxify reactive oxygen species (ROS) (7). VC deficiency is observed in both acute pancreatitis patients and other critically ill patients. Low plasma VC concentration is associated with more severe organ dysfunction and higher mortality (8, 9). A previous study revealed that an oral supplement of VC is slow and produces maximal plasma concentrations that never exceed 220 μM in humans. However, the intravenous supplement of VC is rapid and could reach a concentration of ~100-fold higher than those detected after the oral supplement (10). High-dose intravenous Vitamin C (HDIVC) is a newly developed method for the treatment of critical care diseases, such as sepsis, hemorrhagic shock, and severe burns (11). To date, only three clinical trials investigated the effect of HDIVC on acute pancreatitis. Two studies combined VC with other antioxidants [selenium and N-acetyl cysteine (12) or Vitamin A and Vitamin E (13)], but no significant benefit from the antioxidant therapy was observed. In Du et al.'s study (14), 84 acute pancreatitis patients were recruited, and the results showed that the intravenous VC group had a higher cure rate, a lower complication rate, and a shorter length of stay in hospital compared to the control group. However, over 83% patients recruited in this clinical trial were only affected by mild acute pancreatitis, and the time from the symptom onset to randomization was not clearly clarified. Owing to the lower number of enrollment, combination treatments with other antioxidants, or the mild severity of acute pancreatitis in this trial, the effect of high-dose Vitamin C on the prognosis of SAP is still indeterminate. Our pre-clinical animal study previously reported that HDIVC could alleviate pancreatic injury and improve survival rates (15). Based on these findings and the lack of high-quality clinical evidence to prove the efficiency and safety of HDIVC in the treatment of SAP, we will conduct this single-centered, randomized, double-blinded, placebo-controlled clinical trial to investigate the effect of HDIVC therapy on the prognoses in patients with moderately severe and severe acute pancreatitis.

## Methods and analysis

### Study design

**Figure 1:**
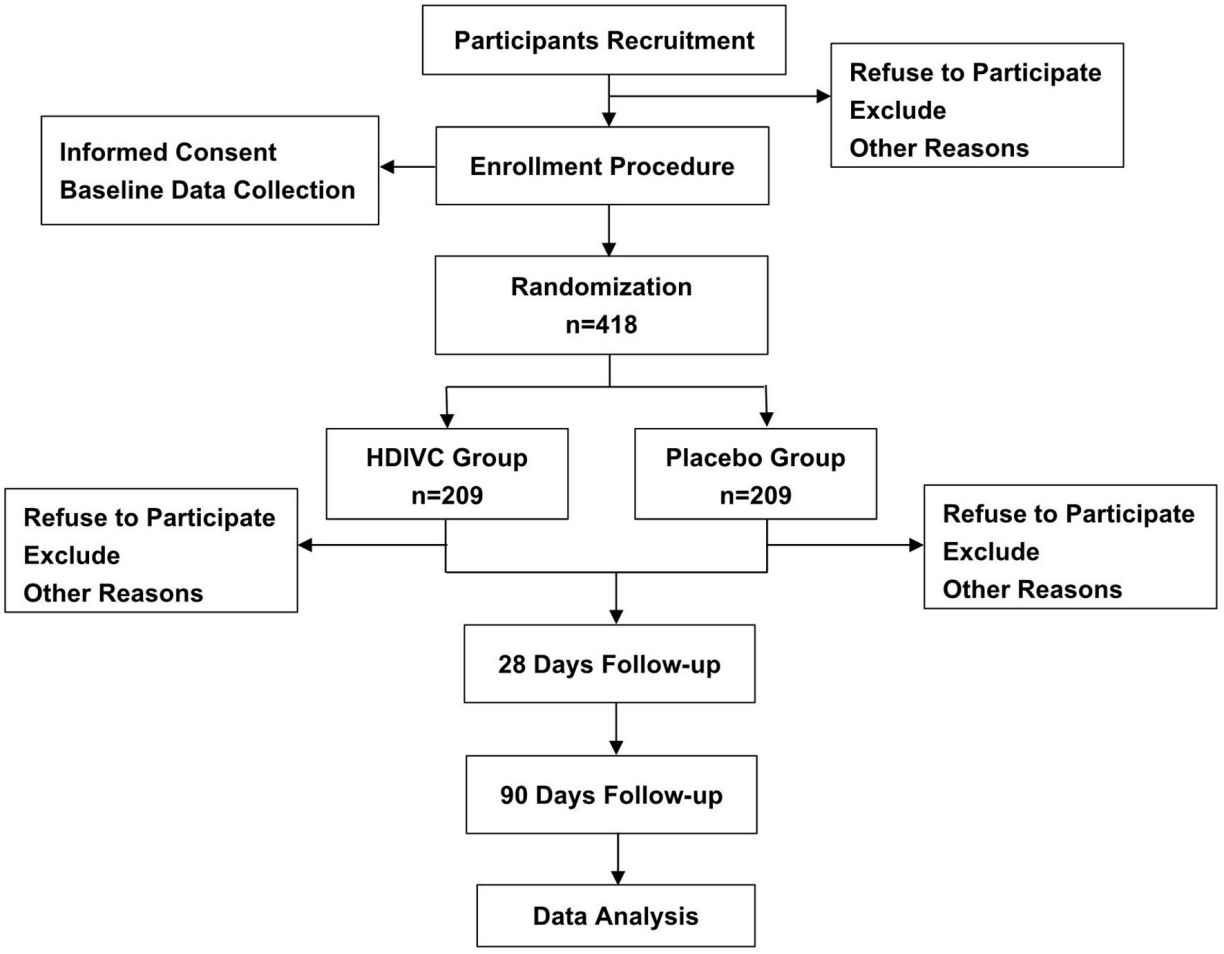
Flowchart of participants.

The study design is a single-centered, randomized, double-blinded, placebo-controlled clinical trial. The study will be conducted in the Department of Emergency, General Surgery and Gastroenterology in Shanghai Ruijin Hospital, Shanghai, China. Eligible patients will be randomly assigned to two groups in a 1:1 ratio to receive treatment with HDIVC (200 mg/kg/24 h) or placebo (saline) for 7 days. The evaluation and examination will be continued from randomization to 90 days or death. Figure 1 shows the flow chart throughout the study. Before enrollment, all participants or legally authorized representatives are required to provide written informed consent. This clinical trial is approved by the institutional ethics board of Ruijin Hospital, Shanghai Jiaotong University School of Medicine. This study is written according to the Standard Protocol Items for Randomized Trials (SPIRIT) statement (16).

### Eligibility criteria

#### Inclusion criteria

The inclusion criteria for the current study are as follows:

▸ patients aged between 18 and 75 years;
▸ time interval from symptom onset to the diagnosis of MSAP or SAP being within 72 h;
▸ those whose clinical conditions, laboratory tests, and imaging examination meet the diagnostic criteria for MSAP and SAP according to the 2012 revised Atlanta guideline (17); and
▸ those who are able to read and write in Chinese and who provide informed consent.

#### Exclusion criteria

The exclusion criteria for the current study are as follows:

▸ acute pancreatitis resulting from a malignant tumor or ERCP;
▸ pregnant or lactating patients;
▸ individuals with poorly controlled chronic organ failure, defined as those who have (1) chronic cardiovascular dysfunction requiring long-term mechanical hemodynamic support or inotrope support, (2) chronic obstructive pulmonary disease requiring home oxygen, (3) chronic hepatic dysfunction classified as Child-Pugh C, (4) chronic renal disease with an eGFR < 60 mL/min/1.73 m^2^ or serum creatinine > 150 μmol/L, and (5) sepsis before enrollment;
▸ individuals with autoimmune diseases or those who have a persistent immunosuppressive state. This includes (1) individuals who have uncured malignant tumors, (2) those who have undergone transplantation status, and (3) those who have been using immunosuppressive agents or hormones for a long time (at least 1 month before the symptom onset). Such agents include steroids (at least 10 mg/day of prednisone or equivalent), calcineurin inhibitors, mTOR inhibitors, unspecific immunosuppressors, TNF inhibitors, or immunosuppressive monoclonal antibodies (4), AIDS, etc.;
▸ individuals with kidney stones;
▸ individuals allergic to experimental drugs;
▸ patients implanted with certain special devices (e.g., pacemakers) that cannot undergo imaging examination; and
▸ patients involved in other clinical trials.

### Intervention

In this study, all the patients will receive regular treatment for acute pancreatitis, such as fluid resuscitation, protease inhibitors, proton pump inhibitors, nutritional support, and antibiotics if infection is suspected. In the HDIVC group, VC with a dose of 200 mg/kg/24 h (15 g/day for a standard 75 kg adult male) will be infused from central venous line for 7 days continuously after randomization. Even though there is no precise definition of the “high dose” of VC, the VC dose in our study is higher than that in Du et al.'s study (10 g/day) (14). We consider this dose to be high and safe, as higher doses of VC have been administered to patients with cancer without significant adverse events (18). In the placebo group, the patients will be infused with the same amount of saline. The administration of study agents will begin at 6 a.m. every day, continuing for up to 7 days, until death, or discharge from the ICU, whichever occurs first.

### Outcomes

Table 1 shows the collected procedures, data, and outcomes.

**Table 1:** Collected procedures, data, and outcomes.

**Procedures**	**Day 0**	**Day 1**	**Day 2**	**Day 3**	**Day 5**	**Day 7**	**Day 14**	**Day 28**	**Day 90**
Eligible criteria	X								
Informed consent	X								
Demographic and medical data	X								
Vital signs	X	X	X	X	X	X	X	X	
Abdominal CT scan	X					X	X	X	
Routine laboratory test^*^	X	X	X	X	X	X	X	X	
Exploratory biomarkers^#^	X					X	X	X	
Plasma vitamin C concentration	X	X	X	X	X	X			
SOFA score	X					X	X	X	
APACHEII score	X					X	X	X	
Organs support						X		X	
Surgical intervention								X	
Vasoactive drugs	X	X	X	X	X	X	X	X	
Death								X	X
Hospital stay									X

^*^Routine laboratory tests include: white blood cell (WBC) count, hemoglobin, thrombocytes, blood gas analysis, electrolytes, urea, creatinine, C-reactive protein (CRP), aspartate transaminase, alanine transaminase, lactate dehydrogenase, alkaline phosphatase, NT-proBNP, TnI, procalcitonin (PCT), coagulation test.

^#^Exploratory biomarkers include: reactive oxygen species (ROS), reactive nitrogen species (RNS), 8-hydroxy-2 deoxyguanosine (8-OHdG), Malondialdehyde (MDA), glutathione (GSH), TNF-a, IL-6, IL-10.

CT, Computed Tomography; SOFA, Sequential Organ Failure Assessment; APACHEII, Acute Physiology And Chronic Health Evaluation II.

#### Primary outcome

This study aims to prospectively assess the effect of HDIVC therapy on 28-day all-cause mortality in patients with moderately severe and severe acute pancreatitis.

#### Secondary outcomes

The secondary outcomes of this study are as follows: (1) organ function indicators: (1) Sequential Organ Failure Assessment (SOFA) scores at 3 and 7 days after admission along with changes in the SOFA scores from Day 0 to Day 3 and Day 7 (19) and (2) the duration of organ support including mechanical ventilation and renal replacement therapy within 3 and 7 days; (2) inflammatory biomarkers: (1) changes in the plasma C-reactive protein on Day 0, Day 3, and Day 7 and (2) ratio of patients with systemic inflammatory reactive syndrome (SIRS) on Day 0, Day 3, and Day 7; and (3) fluid resuscitation: A subgroup of patients who meet the criteria for controlled fluid resuscitation (20) include heart rate (HR) ≥120 beats/min; mean arterial pressure (MAP) ≥85 or ≤ 60 mm Hg; blood lactate concentration (BLC) ≥2 mmol/L; urine output (UO) ≤ 0.5 mL·kg^−1^·h^−1^; and hematocrit (HCT) level ≥44%, and three or more of these parameters combined together. These patients received blood volume expansion as the first part of controlled fluid resuscitation. The specific protocol of controlled fluid resuscitation is shown in the Supplementary material.

(1) the ratio of patients who reach the resuscitation target within 24 h;
(2) fluid amounts that were compared between two groups of patients who met resuscitation targets;
(3) the ratio of patients with MSAP on admission who evolved to SAP; and
(4) 90-day mortality.

#### Sample size

The sample size calculation is based on the primary outcome of 28-day mortality in patients with moderately severe and severe acute pancreatitis. Based on the previously published retrospective research data in our center (21), it was estimated that the mortality rate within 28 days would be 35%. To detect a decrease of 10% mortality rate in the treatment group and 10% dropout rate, 209 patients will be needed in both groups. In total, we plan to recruit 418 patients to be included in the study. All statistical analyses will be performed using two-sided tests with α = 0.05 and 80% power.

### Randomization and blinding

Patients will be randomized in a 1:1 ratio to either the HDIVC group or the control group within 24 h after enrollment. The randomized sequence will be generated using SAS9.4 statistical software (SAS Institute Inc., USA) and sealed in envelopes by a statistician in our clinical research center. The sequence number will be marked outside the envelope and the medication puzzle for Vitamin C or NS will be sealed inside. The randomization information will be blinded to all the participants in the trial except the unblinding drug preparing nurse (UDPN). Once consent is obtained and inclusion and exclusion criteria are verified, the PI will hand the unique randomized envelope of this patient to the UDPN. The UDPN will unseal the envelope and fill a 50-ml syringe with either Vitamin C or NS based on the grouping information inside the envelope. The UDPN will give the opaque syringe to the therapy nurse and will not participate in the subsequent medical processes. The outcome assessor and statistician will be blinded to randomization and will not be involved in treatment procedures. Unblinding is permissible if severe adverse events occur or participants need to withdraw from the trial, and the statistician will be consulted to provide the necessary information.

### Data collection and management

The data will be collected from case report forms by authorized assessors. When participants withdraw from the study, they are not replaced. Patients who are lost during the study period will be incorporated into the analysis until their final leaving date. Missing data will be replaced by chained equations using multiple imputations if these missing data are judged to be random. If the missing data are non-random, then the technique of the last observation carried forward (LOCF) will be adopted to handle the missing data.

### Statistical methods

Categorical data will be presented with frequency or ratio, while continuous variables will be described using the mean and standard deviation (SD) for normally distributed variables, or median and interquartile range (IQR) for non-normally distributed variables. Categorical variables will be compared using the χ^2^-test or Fisher's exact test. Continuous variables will be compared using the *t*-test for normally distributed variables or the Wilcoxon rank-sum test for non-normally distributed variables. The primary analysis will be conducted using the intention-to-treat (ITT) approach analyses. The multivariate stepwise logistic regression model will be adopted to address potential confounding variables. The interim analysis will be performed after the inclusion of 200 patients. A two-sided *p*-value lower than 0.05 will be considered statistically significant. All statistical analyses were performed using SPSS (Version 19.0).

The primary outcome of 28-day mortality between the HDIVC group and placebo group will be compared using the χ^2^-test.

The statistical methods of the secondary outcomes are shown in Supplementary Table 1.

### Adverse event reporting

An adverse event (AE) refers to “any untoward medical occurrence in a human subject, including any abnormal sign (e.g., abnormal physical exam or laboratory finding), symptom, or disease” that occurs during a subject's participation in research (22).

AEs include

(1) crystalluria,
(2) respiratory failure,
(3) thromboembolic disease,
(4) arrhythmias,
(5) delirium,
(6) anemia, and
(7) coagulopathy.

Severe AEs (SAEs) include

(1) unexplained acute kidney failure,
(2) death,
(3) life-threatening conditions,
(4) prolongation of the existing hospitalization, and
(5) persistent or significant disability/incapacity.

Both AEs and SAEs will be evaluated at 8:00 a.m. every day. The adverse events will be evaluated according to the CTVAE V5.0 (Common Terminology Criteria for Adverse Events) issued by the U.S. Department of Health and Human Services. They will be recorded and reported to the PI of each research site and evaluated if they are related to this study. Once confirmed, the given medicine should be discontinued immediately. The AE-related indicators as well as its development should be monitored closely. Both AEs and SAEs will be summarized.

### Monitoring

To standardize the research procedures, the researchers, pharmacists, physicians, and nurses are trained in standard clinical practice. A manual of evaluations and a case report form will be developed for every participant. Obtained data will be stored with utmost security and confidentiality. Data will be analyzed by a statistician after the removal of any participant identifier information and the analysis of the data will occur only upon the completion of this study.

## Dissemination plan

Data will be published in peer-reviewed journals and presented at conferences, both nationally and internationally. The original data will be revealed on the website of ResMan after the completion of the trial, http://www.medresman.org.cn.

## Trial status

The trial was started on September 1, 2019. Considering the influence of COVID-19, the anticipated recruitment end date has been extended to December 31, 2023.

## Ethics statement

This clinical trial is approved by the Biomedical Research Ethics Committee of the Ruijin Hospital, Shanghai Jiao Tong University School of Medicine (Registration Number: 2019-90).

## Author contributions

JL: Writing—review & editing, Writing—original draft. BZ: Writing—review & editing, Writing—original draft. WS: Writing—original draft, Writing—review & editing. YW: Writing—review & editing. XQ: Writing—review & editing. NN: Writing—review & editing. SS: Writing—review & editing. ML: Writing—review & editing. YY: Writing—review & editing. TN: Writing—review & editing. LM: Writing—review & editing. JHe: Writing—review & editing. JHu: Writing—review & editing. ZY: Writing—review & editing. YC: Writing—review & editing. HS: Writing—review & editing. EM: Writing—review & editing.
