# Supplementary material for: Effect of high-dose intravenous vitamin C therapy on the prognosis in patients with moderately severe and severe acute pancreatitis: protocol of a prospective, randomized, double-blinded, placebo-controlled study
Source: Front Med (Lausanne). 2023 Nov 9;10:1278167. doi: 10.3389/fmed.2023.1278167 (PMC10665842; doi:10.3389/fmed.2023.1278167)
Supplement: Supplementary file 1 [file Data_Sheet_1.docx]

**Controlled** **fluid** **resuscitation[1]**

**Indications**

Controlled fluid resuscitation is only suitable for patients with severe blood volume deficiency within 72 h after onset and three or more of the following criteria: heart rate (HR) ≥ 120 beats/min; mean arterial pressure (MAP) ≥ 85 mmHg or ≤ 60 mmHg; blood lactate concentration (BLC) ≥2 mmol/L; urine output (UO) ≤0.5 mL ·kg- 1 ·h- 1; and hematocrit (HCT) level ≥44%.

**Blood** **volume** **expansion**

At a crystal-to-colloid ratio of 2:1, infusion is performed using two vascular pathways simultaneously. If MAP is <60 mmHg, then it must be increased to >60 mmHg using a pressor agent and rapid infusion within 30 min, followed by controlling the optimum infusion rate (5– 10 mL/kg/h). The total amount of infusion fluid should be controlled by evaluating it every 4 h, even if the blood volume expansion has reached the required standard. Blood volume expansion was considered to have reached the standard as long as it occurred slowly, within 24 h after admission, and when two or more of the following requirements were met: MAP 65 to 85 mmHg; normal urine volume; or HCT 30% to 35%.

**Adjustment** **of** **body** **fluid** **distribution**

After the blood volume expansion reached the required standard, the body fluid distribution should be quickly adjusted; excess liquid infused during the expansion phase should be excreted from the body or the continual loss of body fluid should be resuscitated. Infusion fluid is primarily composed of colloids and crystals (colloid-to- crystal ratio of 3:1) that have diuretics and/or continuous renal replacement therapies added to them.

**Endpoint** **of** **fluid** **resuscitation**

The gold standard for determining the endpoint of fluid resuscitation is the disappearance of oxygen debt. However, the determination of oxygen debt requires technology such as floating catheters or pulse contour cardiac output, which limits routine clinical applications. Therefore, it is imperative to use simple clinical indicators to determine the endpoint of liquid resuscitation. We propose that the disappearance of SIRS should be the endpoint of fluid resuscitation for SAP because the distance between capillaries becomes normal and oxygen intake returns to normal, so as to eliminate oxygen debt, only after the disaparance of SIRS; however, this view has not yet been widely accepted.

**Reference**

1. Mao E. Intensive management of severe acute pancreatitis. Ann Transl Med. 2019;7:687.

**Supplementary table 1. List of outcomes and statistical methods.**

| Outcomes | | | | Statistical methods |
| --- | --- | --- | --- | --- |
| Primary outcome | 28 days mortality (all-cause mortality) |  |  | χ2 test |
| Second outcome | Organ function indicators | Sequential Organ Failure Assessment (SOFA) scores |  | Τ-test or Wilcoxon rank-sum test |
|  |  | The duration of organ support | Mechanical ventilation | Τ-test or Wilcoxon rank-sum test |
|  |  |  | Renal replacement therapy |  |
|  | Inflammatory biomarkers | Changes in the plasma C-reactive protein |  | Τ-test or Wilcoxon rank-sum test |
|  |  | Ratio of patients with systemic inflammatory reactive syndrome (SIRS) |  | χ2 test or Fisher exact test |
|  | Fluid resuscitation* | The ratio of patients who reach the resuscitation target within 24 hours. |  | χ2 test or Fisher exact test |
|  |  | Among the patients who reach the resuscitation target, fluid amounts between two groups were compared. |  | t-test or Wilcoxon rank-sum test |
|  | Ratio of patients with MSAP on admission who evolved to SAP |  |  | χ2 test or Fisher exact test |
|  | 90 days mortality |  |  | χ2 test or Fisher exact test. Kaplan-Meier methods and log-rank test. |

*A subgroup of patients who meet the indications for controlled fluid resuscitation as following criteria: heart rate (HR) ≥120 beats/min; mean arterial pressure(MAP) ≥85 mm Hg or ≤60 mm Hg; blood lactate concentration (BLC) ≥2 mmol/L; urine output (UO) ≤0.5 mL·kg-1·h-1; and hematocrit (HCT) level ≥44%, three or more are met. These Patients receives blood volume expansion as first part of controlled fluid resuscitation. The specific protocol of controlled fluid resuscitation is shown in **supplementary**.

**Supplementary table 2. List of vital signs.**

| Vital signs | Temperature |
| --- | --- |
|  | Systolic blood pressure |
|  | Diastolic blood pressure |
|  | Pulse rate |
|  | Breath rate |
|  | Blood oxygen saturation |
|  | Central venous pressure |

Vital signs are collected on day 0, day 1, day 2, day 3, day 5, day 7, day 14, day 28, and day 90.
